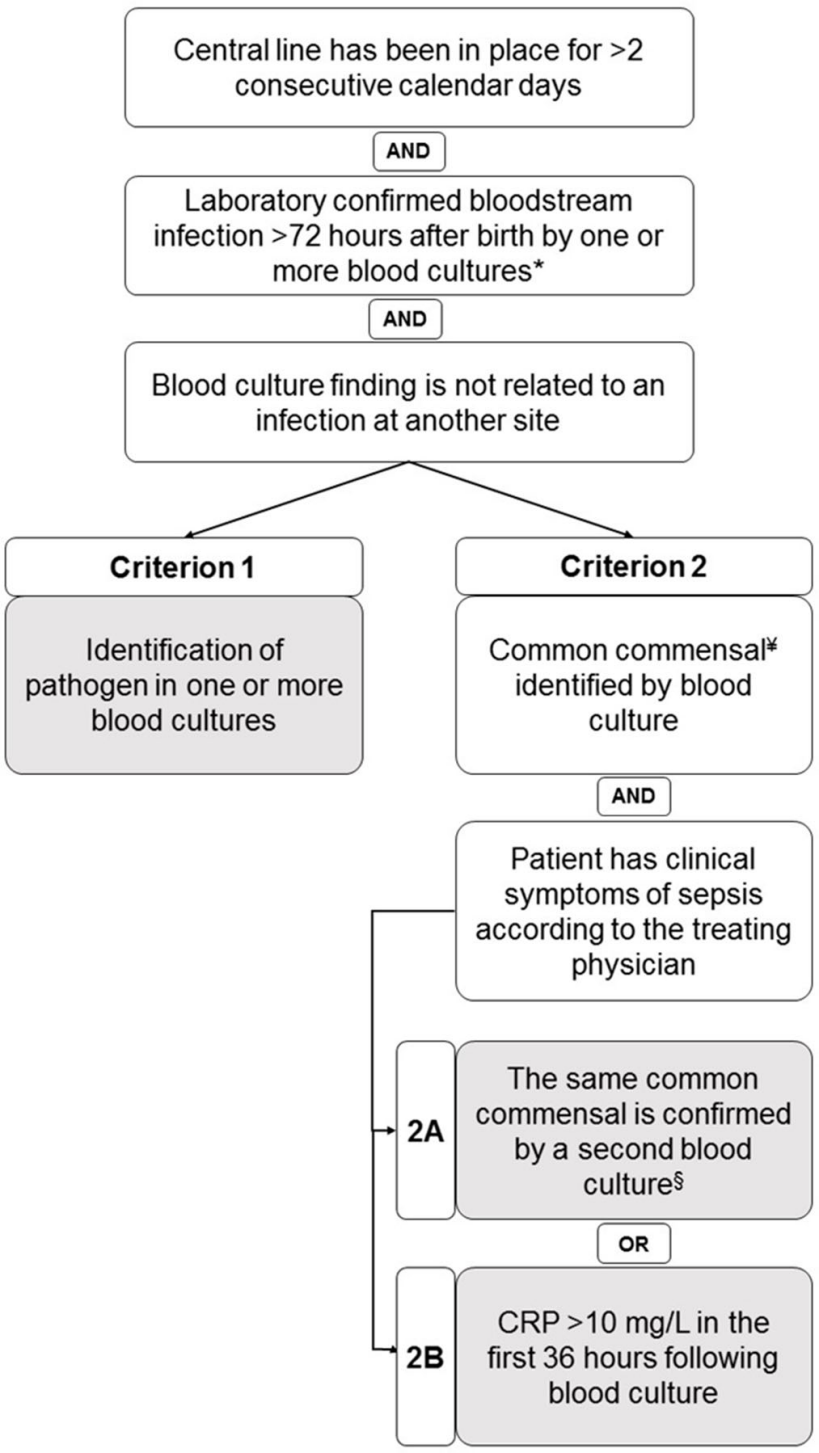# Sustainable Neonatal CLABSI Surveillance: Consensus Toward New Criteria in the Netherlands

**DOI:** 10.1017/ash.2021.14

**Published:** 2021-07-29

**Authors:** Ilja Heijting, Joost Hopman, Marije Hogeveen, Willem de Boode, Alma Tostmann, Tim Antonius

## Abstract

**Group Name:** Working Group on Neonatal Infectious Diseases of the Section of Neonatology of the Dutch Paediatric Society

**Background:** Central-line–associated bloodstream infections (CLABSIs) are a main focus of infection prevention and control initiatives in neonatal care. Standardized surveillance of neonatal CLABSI enables intra- and interfacility comparisons, which can contribute to quality improvement. To date, there is no national registration system for CLABSI in neonatal care in the Netherlands. Across neonatal intensive care units (NICUs), several different sets of CLABSI criteria and surveillance methods are used for local monitoring of CLABSI incidence rates. To achieve standardized CLABSI surveillance, we conducted a consensus procedure with regard to nationwide neonatal CLABSI surveillance criteria. **Method:** A modified Delphi consensus procedure for the development of nationwide neonatal CLABSI surveillance criteria was performed between January 2016 and January 2017 in the Netherlands. An expert panel was formed by members of the Working Group on Neonatal Infectious Diseases of the Section of Neonatology of the Dutch Paediatric Society. The consensus procedure consisted of 3 expert panel rounds. Figure 1 shows a detailed description of the consensus procedure. **Result:** The expert panel achieved consensus on Dutch neonatal CLABSI surveillance criteria, which are summarized in Figure 2. Neonatal CLABSI is defined as a bloodstream infection occurring >72 hours after birth, associated with an indwelling central venous or arterial line and laboratory confirmed by 1 or more blood cultures. In addition, the blood culture finding should not be related to an infection at another site and one of the following criteria can be applied: (1) a bacterial or fungal pathogen is identified from 1 or more blood cultures; (2) the patient has clinical symptoms of sepsis and (2A) a common commensal is identified in 2 separate blood cultures or (2B) a common commensal is identified by 1 blood culture and C-reactive protein (CRP) level is >10 mg/L in the first 36 hours following blood culture collection. **Conclusion:** The newly developed Dutch neonatal CLABSI surveillance criteria are concise, are specific to the neonatal population, and comply with a single blood-culture policy in actual neonatal clinical practice. International agreement upon neonatal CLABSI surveillance criteria is needed to identify best practices for infection prevention and control.

**Funding:** No

**Disclosures:** None

**Figure 1.**
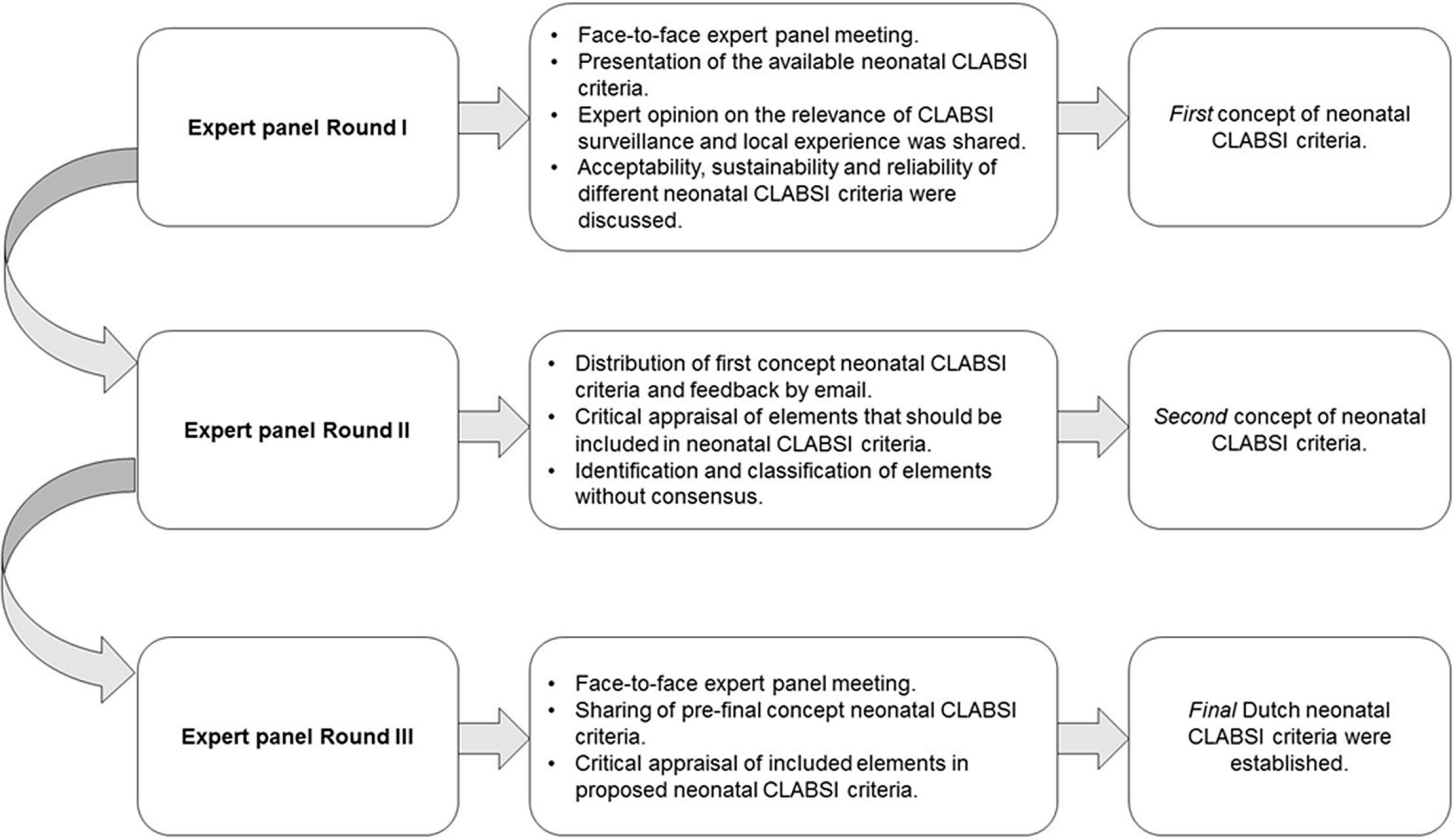

**Figure 2.**